# A treponemal genome from an historic plague victim supports a recent emergence of yaws and its presence in 15^th^ century Europe

**DOI:** 10.1038/s41598-020-66012-x

**Published:** 2020-06-11

**Authors:** Karen Giffin, Aditya Kumar Lankapalli, Susanna Sabin, Maria A. Spyrou, Cosimo Posth, Justina Kozakaitė, Ronny Friedrich, Žydrūnė Miliauskienė, Rimantas Jankauskas, Alexander Herbig, Kirsten I. Bos

**Affiliations:** 1 Max Planck Institute for the Science of Human History, Jena, Germany; 2 Vilnius University, Vilnius, Lithuania; 3 Curt-Engelhorn-Zentrum Archäometrie, Mannheim, Germany; 4 Institute for Archaeological Sciences, Archaeo- and Palaeogenetics, University of Tübingen, Rümelinstraße 23, 72070 Tübingen, Germany

**Keywords:** Genome, Microbial genetics, Microbiology, Bacteria, Microbial genetics, Pathogens

## Abstract

Developments in techniques for identification of pathogen DNA in archaeological samples can expand our resolution of disease detection. Our application of a non-targeted molecular screening tool for the parallel detection of pathogens in historical plague victims from post-medieval Lithuania revealed the presence of more than one active disease in one individual. In addition to *Yersinia pestis*, we detected and genomically characterized a septic infection of *Treponema pallidum pertenue*, a subtype of the treponemal disease family recognised as the cause of the tropical disease yaws. Our finding in northern Europe of a disease that is currently restricted to equatorial regions is interpreted within an historical framework of intercontinental trade and potential disease movements. Through this we offer an alternative hypothesis for the history and evolution of the treponemal diseases, and posit that yaws be considered an important contributor to the sudden epidemic of late 15^th^ century Europe that is widely ascribed to syphilis.

## Introduction

Ancient DNA analyses have the potential to reveal history that is hidden within archaeological samples. Increased sequencing capacity made available by technological innovations has translated into concomitant increases in temporal depth for human genomes^1^ as well as those from commensal^2^ and invasive^3^ microbes. Genome-level analyses of ancient bacteria and viruses have revealed unexpected patterns of pathogen dispersal in the past, and have prompted discussants to revise long standing theories about the evolution of some of our most well-studied diseases such as plague^4^, tuberculosis^5^ and smallpox^6^ to name but a few.

Genomic investigations of ancient microbes have largely been made possible via selective enrichment for DNA of a target organism. By design, this process requires *a priori* knowledge of a taxon of interest, which is typically acquired through either initial molecular screening^7^, inference from historical context^8^, diagnostic gross pathology^9^, or combinations thereof^5^. More recent approaches have explored the analytical resolution of broad multi-species enrichments^10^ or fully non-targeted approaches^11,12^ for pathogen detection. Though demonstration of these techniques is currently restricted to single microbial organisms, they indeed offer the flexibility to detect co-morbidities on historical timescales, thus giving a glimpse into past disease ecology.

Here we present the application of an ancient pathogen detection method to identify co-morbidities in archaeological specimens associated with an historical epidemic. The influence of co-infections as exacerbating the progression of certain diseases is an established phenomenon^13,14^ and has been proposed to partially account for plague’s unusually high mortality in the past^15,16^, its potential influence on selective mortality^16^, as well as its persistence and subsequent rapid extinction in post-medieval Europe^8^. As a first investigation into the existence of co-morbidities in post-medieval plague victims we applied a non-targeted pathogen screening approach offered through the HOPS platform^17^ to skeletal material from a suspected 15^th^–16^th^ century plague burial from Vilnius, Lithuania. HOPS couples high sensitivity in taxon assignment with an evaluation of molecular qualities common to ancient DNA: together these features make it an ideal tool for detection of concurrent infections in metagenomic data from archaeological tissues. In addition to *Yersinia pestis*, our analyses succeeded in identifying a disease of the treponemal family, *Treponema pallidum pertenue* (yaws), in one of the four confirmed plague victims. While a physiological relationship between the two infections is not explored here, genomic data from both pathogens are considered independently in terms of their unique evolutionary histories. Analyses of the newly reconstructed treponemal genome led us to propose a novel hypothesis that a recent introduction of yaws may have contributed to the devastating post-medieval outbreaks that are commonly attributed to the onset of syphilis in Europe.

## Site Description

**Figure 1:**
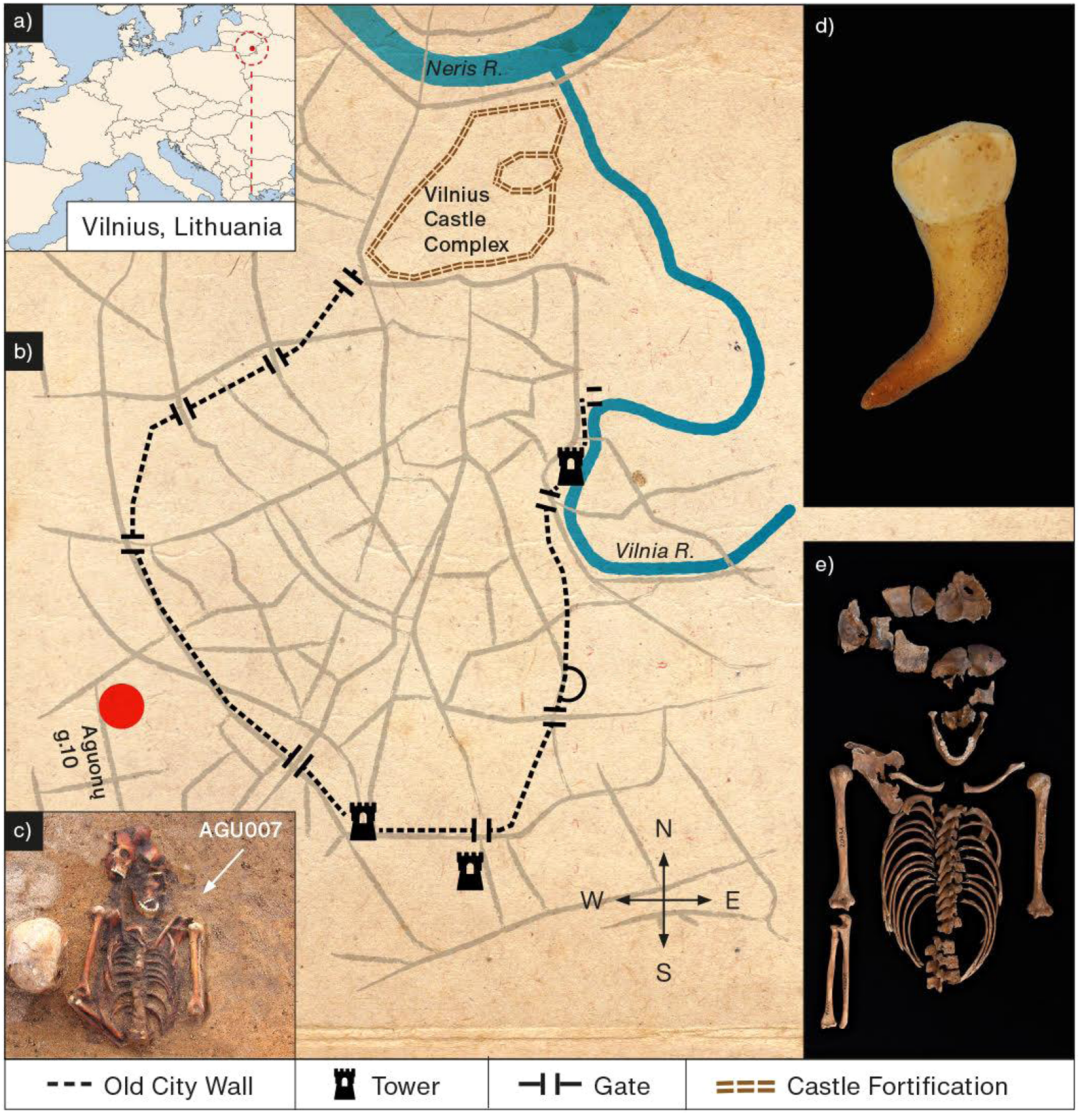
(**a,b**) Map showing the city of Vilnius, Lithuania, and the relative positions of the castle fortification, the ancient city wall, and the archaeological site, Aguonu g. 10 (red circle). (**c**) Multiple burial containing individual AGU007, indicated by the white arrow (photograph courtesy of Robertas Žukovskis). (**d**) Premolar from individual AGU007 used for molecular analyses. (**e**) exhumed skeleton of individual AGU007 (photograph courtesy of Justina Kozakaitė). Figure created by Hans Sell.

In 2006 and 2007, a post-medieval cemetery containing 119 burials and 216 individuals was excavated in Vilnius, Lithuania. As historical or cartographic descriptions of the site are lacking, its existence was unknown prior to excavation. The cemetery is located outside the boundary of the medieval city walls (Fig. 1), and contained a number of multiple burials – 33 of these contained up to 15 individuals in a single grave, and are thus interpreted to each represent single mortality events^18^. These contexts, as well as the demographic composition that differs from other attritional contexts (here with young adults and adolescents prevailing), led to the site’s recognition as a potential burial ground for victims of epidemics^18^.

## Results

### Pathogen screening and genomic reconstruction

Powdered material from the pulp chamber of one tooth from each of 26 individuals and their associated negative controls were processed following methods described elsewhere (Supplementary Information) in the dedicated ancient DNA facility of the Max Planck Institute for the Science of Human History. To identify plague victims in the skeletal series, presence of *Yersinia pestis* DNA in the extract was assessed via a targeted amplification-based quantitative PCR (qPCR) approach for the pestis-specific *pla* gene of the PCP1 plasmid^19^. This assay revealed six putative plague victims: individuals AGU003, AGU009, AGU010, AGU013, AGU020 and AGU025 (Table S1; Figures S1; S2). Negative controls were free of amplification products.

In a separate strategy, double-stranded Illumina screening libraries were prepared for all 26 individuals from 10 µL of DNA extract following established protocols^20^. Double-stranded libraries from 40 µL of DNA extract were prepared alongside for the six putative plague victims, with enzymatic treatment to remove taphonomic chemical damage in the form of cytosine deamination^21^. All libraries were subsequently processed following established protocols^22^ and were sequenced on an Illumina HiSeq 4000 to a depth of ca. 10 million reads each. Amplified UDG-treated and untreated libraries of the putative plague victims, as well as their associated negative controls, were prepared for two consecutive rounds of in-solution whole genomic *Y. pestis* capture performed as previously described^23^. Samples were captured in individual wells, with negative controls pooled in a single capture reaction. Capture products were sequenced to a depth of ca. 10 million reads each on an Illumina HiSeq 4000. All negative controls were sequenced on a NextSeq 500 to a depth of ca. 2 million reads each.

Mapping of the sample screening (i.e. shotgun) libraries to the *Y. pestis* CO92 reference genome (NC_003143.1)^24^ yielded between 2 and 87 unique *Y. pestis* chromosomal fragments for each screening library after duplicate removal (Table S3). At most 30 chromosomal fragments were detected in any one of our negative controls. Unexpectedly, the greatest number of mapping reads was identified in individual AGU007: this sample performed poorly in our initial *Y. pestis* qPCR screen despite meticulous evaluation of PCR inhibition prior to *pla* amplification (Figure S1, Table S1). For authentication, a non-UDG library from this individual was prepared for two rounds of in-solution *Y. pestis* capture and sequenced on a NextSeq 500. A UDG-treated library was also prepared, shotgun sequenced, and captured for *Y. pestis*, all carried out as described above with sequencing done on an Illumina HiSeq 4000.

Enriched products yielded convincing signals of ancient *Y. pestis* preservation in individuals AGU007, AGU010, AGU020, and AGU025 (Table S4, Table S5, Figure S4), though at levels too low to permit genomic reconstruction. This motivated us to process a second tooth from each of these individuals with the goal of increasing genomic coverage. DNA from these additional teeth was extracted and shotgun sequenced, as well as captured and sequenced to a depth of approximately 20 million reads following methods described above. Due to the low coverage of individual AGU020 (Table S4), an additional UDG library aliquot for the first tooth was captured along with this second set. Enriched reads from both AGU020 UDG-treated fractions from the first tooth were merged for analysis. Subsequent mapping to the *Y. pestis* reference after duplicate removal yielded mean coverages of 1.68- to 38.01-fold for all UDG-treated fractions, with the highest amounts identified in individual AGU007, which also showed excellent preservation of human DNA (Table 1, Tables S6-S10). Signals of ancient damage were detected in all untreated libraries (Figure S4). At most 24 reads mapped to the *Y. pestis* chromosome in our negative controls (Table S8).

**Table 1:** Mapping statistics for *Y. pestis* and *T. pallidum* capture products. Results are reported for the second processed tooth only unless otherwise specified.

Individual	Library Type	Raw reads	Mapping Reads post duplicate removal	Target DNA (%) post enrichment	Mean Fold Genomic Coverage	Percentage of Genome Covered at 3X(%)
**a)** ***Y. pestis*** **mapping:**						
AGU007	non-UDG	20901682	1704427	36.22	26.81	94.10
	UDG	23998414	2344695	37.33	38.01	94.32
AGU010	non-UDG	24499218	970324	30.18	12.52	92.57
	UDG	24725162	1480281	25.45	20.76	93.51
AGU020	*merged	14397991	132259	2.55	1.68	23.66
	non-UDG	19136670	3978	0.19	0.04	0.06
	UDG	19925684	8235	0.17	0.09	0.09
AGU025	non-UDG	19964532	266287	23.19	4.17	74.30
	UDG	23621324	849774	24.94	14.36	93.65
**b)** ***T. pallidum*** **mapping:**						
AGU007	non-UDG	46614028	146398	7.64	9.31	96.68
	UDG	45771712	330505	8.10	21.36	97.69

*Merged data from 2 aliquots of the UDG library of the first tooth.

To investigate whole microbial content, shotgun libraries from the initial screening dataset for the 26 teeth were evaluated using the MEGAN Alignment Tool (MALT) and queried for the presence of pathogen DNA via the screening pipeline HOPS^17,25^. The MALT database was constructed from a custom RefSeq Genome set in November 2017 that contained bacteria, viruses, and eukaryotes (ftp://ftp.ncbi.nlm.nih.gov/genomes/refseq/). Predictably, *Y. pestis* signals were detected in both the UDG-treated and untreated libraries for individuals AGU007, AGU010, AGU020 and AGU025, all of which showed evidence of DNA damage (Figure S4). In further contrast to the qPCR screening results, no *Y. pestis* signals were detected in either the UDG-treated or untreated libraries for individuals AGU003, AGU009, and AGU013, indicating that these individuals may have yielded false positive identifications in the initial qPCR screen.

**Figure 2:**
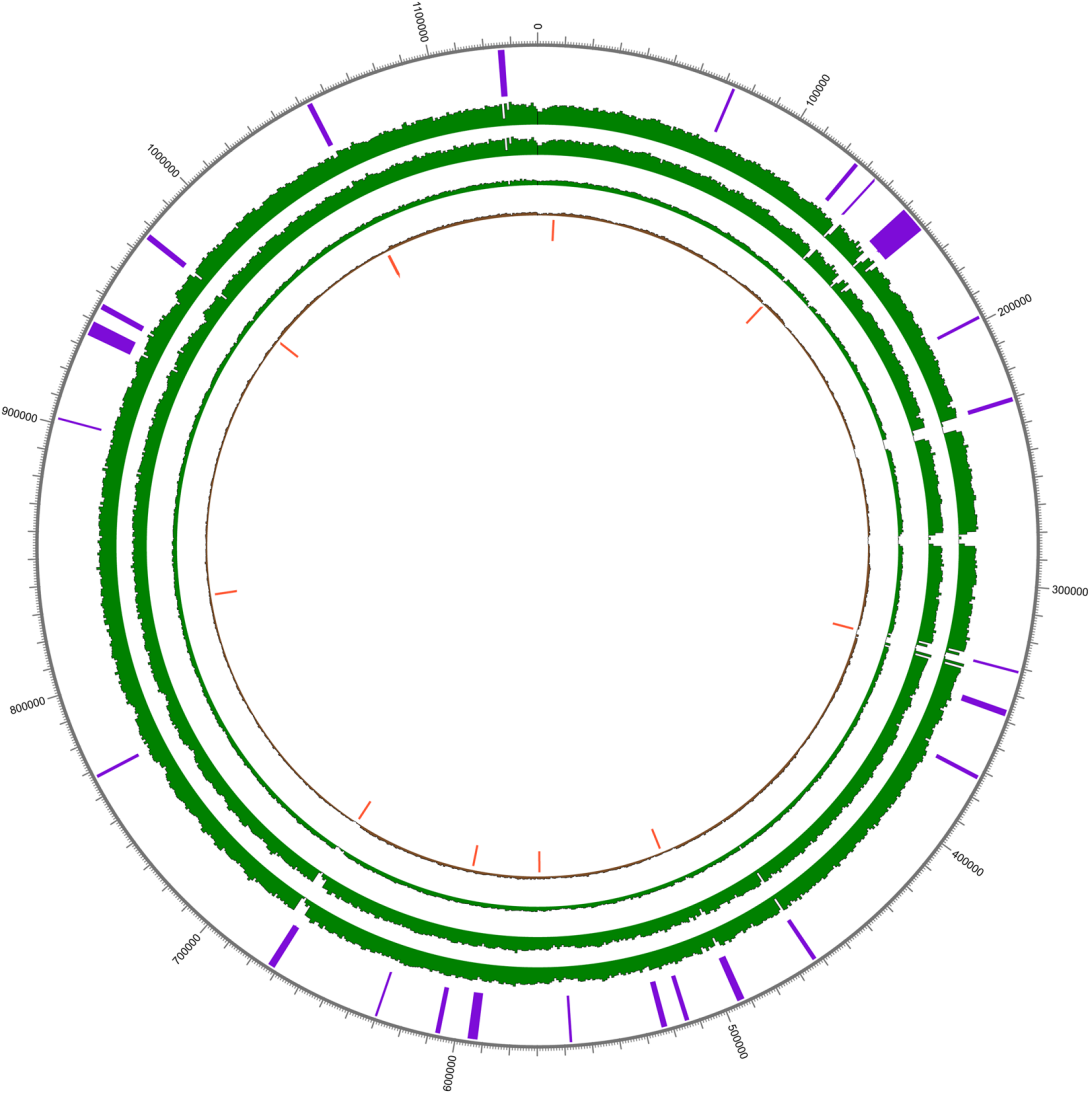
Circos coverage plot for ancient *Treponema pallidum pertenue*. The innermost circle represents 13 homoplastic sites (orange) detected between *pallidum, pertenue* and *endemicum* strains through SNP evaluation. Histogram showing the coverage of ancient *pertenue* genome 133 (Schuenemann *et al*., 2018) is shown in brown. Coverages of enriched data for AGU007 are shown in green for first enrichment (inner), second enrichment (middle) and merged set (outer). The outermost positions in purple highlight the recombining regions identified by ClonalFrameML.

Individual AGU007, however, showed a weak signal for *Treponema pallidum*, a spirochete bacterium associated with the human diseases pinta, bejel, yaws, and most famously syphilis. Though its identification in HOPS was limited to only 12 assigned reads in the UDG treated library (Figure S3), a close examination of data from the untreated library revealed 7 reads assigned to *T. pallidum*, with additional reads assigned to *Treponema* at the genus level for both libraries. Mapping against the Nichols reference for *T. pallidum pallidum* yielded 36 and 27 reads in the treated and untreated libraries, respectively (Table S11, Figure S3). Intrigued by this, we captured both the UDG-treated and untreated libraries for individual AGU007 for *T. pallidum* DNA as previously described^9^. *T. pallidum*-enriched libraries were mapped to the Nichols reference yielding 31,882 and 126,552 unique reads and 1.78 and 6.67 mean fold coverage for the untreated and treated fractions, respectively, following duplicate removal (Table S12). The untreated library showed a C to T transition pattern comparable to that from the *Y. pestis* mapping, consistent with chemical damage of ancient molecules (Figures S4 and S5). *T. pallidum* capture of the second tooth from individual AGU007 yielded 330,505 mapping reads (following duplicate removal) that assembled to build a high-quality genome of 21-fold mean coverage (Table S14). Ancient damage patterns observed in the untreated fraction support its authenticity (Table S14; Figure S5). By contrast, the 8 negative controls associated with this capture together yielded only one read in each of three blank libraries that mapped to the reference (Table S14) indicating exceptionally clean working conditions. A BLASTn^26^ evaluation of each read revealed them to come from regions that are conserved in many bacterial taxa, and are hence likely to derive from non-target sources. The two UDG-treated enrichment datasets were subsequently merged to yield a *T. pallidum pertenue* genome of 26-fold mean coverage (Fig. 2).

### Morphological assessment of individual AGU007

The postcranial skeleton of individual AGU007 is extremely fragmented. Morphological analysis was therefore limited to the skull, claviculae, the right scapula, the upper limbs, ribs, and thoracic vertebrae. Periosteal lesions were identified as active when the affected portion of the bone appeared porous with unremodeled edges. Lesions were identified as healed if the affected portion of bone demonstrated rounded and remodeled edges. Individual AGU007 had mixed (active and healed) periosteal lesions on the distal portion of the right humerus and the proximal portion of the right ulna. A slight active periosteal reaction was observed on small fragments of the internal surface of the parietal bones. Images of pathological bone are shown in Figure S6. Pathological surveys are not reported for the remaining individuals of the skeletal series since they yielded no detectable infections other than *Y. pestis*, which does not cause skeletal involvement.

### Human DNA analysis of individual AGU007

**Figure 3:**
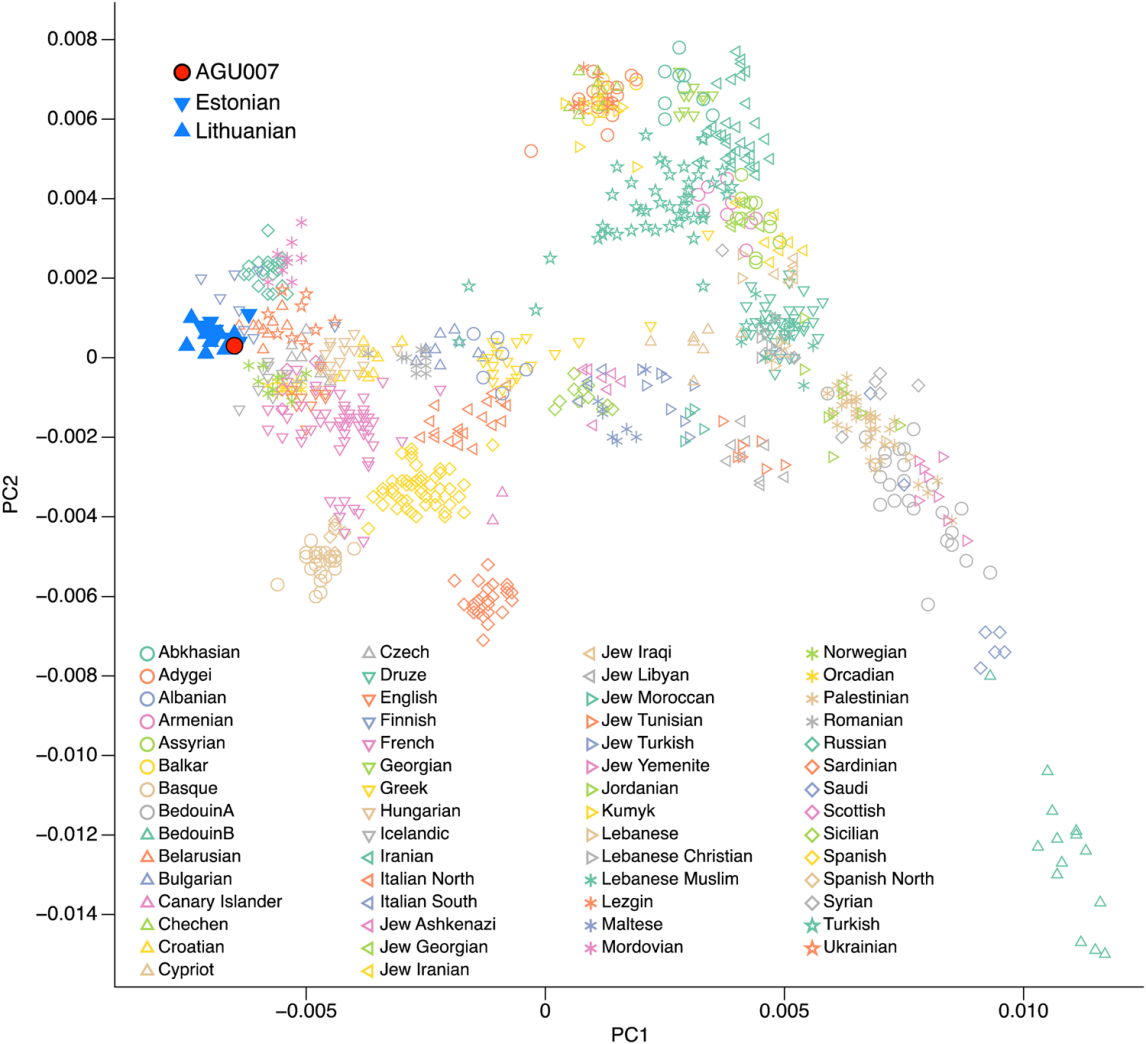
Principal component analysis based on 96,953 SNPs queried against the Human Origins dataset.

*T. pallidum pertenue* is currently restricted to equatorial regions. To determine if individual AGU007 had ancestry associated with areas of modern yaws epidemiology, we undertook human genetic analyses. Shotgun sequencing data from both untreated and UDG-treated libraries for the second tooth of individual AGU007 were mapped against the human reference genome (hg19) with BWA^27^ as implemented in EAGER^28^, and the damage pattern was estimated with *mapDamage2.0*^29^ (Table S6). Based on coverage of the sex chromosomes relative to the autosomes the individual was determined to be female (Table 2a). Untreated sequencing data were also mapped against the mtDNA reference sequence (rCRS) (Table 2b). The resulting alignment was provided as input to *schmutzi*^30^ and the reconstructed mtDNA consensus sequence was assigned to haplogroup H1ap1 using *Haplofind*^31^. MtDNA contamination level was estimated to be ca. 1% (0–2%), which can also be considered a proxy for nuclear contamination due to the low mitochondrial to nuclear DNA ratio (Table 2a)^32^. Sequencing data from the UDG-treated library was subsequently used for pseudo-haploid genotyping with *pileupCaller* (https://github.com/stschiff/sequenceTools) and then merged to the *Human Origins* dataset^33^ resulting in 96,953 overlapping SNPs. A principal component analysis (Fig. 3) was calculated based on modern-day West Eurasian populations^34^ onto which the ancient individual was projected using *smartpca*^35^. This analysis places her genome in close proximity to present-day Baltic populations such as Estonians and Lithuanians.

**Table 2:** Human DNA mapping statistics.

Library type	Raw Reads	Mapped Reads	Target DNA (%)	Mt/nuc Ratio	C to T 5′end (%)	Average fragment length (bp)	X/Y rate
***a) nuclear genome***							
non-UDG	12,853,927	7,492,473	69.99	147.81	16	62.02	0.84/0.04
UDG	14,325,984	9,752,652	68.98	127.29	1	61.29	0.83/0.05
***b) mitochondrial genome***							
Mean Coverage	C to T 5′end (%)	MtDNA Haplogroup	Contamination (%)				
26.80	21	H1ap1	1 (0–2)

### Phylogenetic analyses

#### *Yersinia pestis*

**Figure 4:**
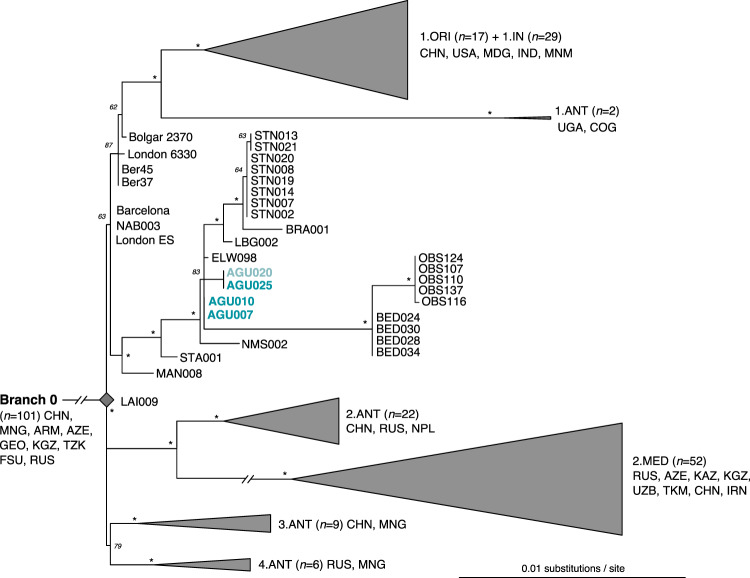
Maximum likelihood tree of post-Black Death genomes of *Y. pestis*. Constructed from 275 genomes with the Generalised Time Reversible (GTR) model, SNPs identified at 3-fold coverage, 1000 bootstrap replicates, and a 98% partial deletion filter (considering 5801 SNPs). Bootstraps with a value of 95 or greater are indicated with an asterisk (*), and all those lower than 95 are presented with their numeric value. A phylogeny showing all genomes considered in this analysis is presented in Figure S7. The extrapolated position for the low coverage genome from individual AGU020 is shown in faded text.

Phylogenetic analyses for *Y. pestis* were carried out using UDG treated datasets obtained from the second tooth only, with the exception of individual AGU020: inclusion of the ca. 8000 mapping reads from the second tooth did not increase its coverage beyond 1.68-fold, and its persistent low recovery led to its exclusion from phylogenetic tree construction. Its tentative position in the tree was rather determined based on manual evaluation of SNPs (Fig. 4, Table S16).

*Y. pestis* SNPs were called using UnifiedGenotyper in the Genome Analysis Toolkit (GATK)^36^ implemented in EAGER, and analyzed within a published set of 275 genomes, of which 41 were ancient (Table S15). Excluded genomic regions were consistent with those selected in other published works^22^. SNPs were identified in all positions that had at least 3-fold coverage, a minimum genotyping quality of 30, and consensus calling at 90% support. This produced a final SNP alignment of 6949 positions (Table S16). From this, a maximum likelihood phylogenetic tree was constructed with RAxML^37^ (Figs. 4 and S7).

All Lithuanian *Y. pestis* genomes share a number of positions that place them within the diversity of the second pandemic descendants of the Black Death (London ES, NAB003, and Barcelona 3031) that are hypothesised to represent plague’s post-Black Death persistence in Western Europe. Based on our current phylogeny, two of these genomes, AGU007 and AGU010, are most closely related to the strain from Ellwangen, Germany^8^, here identified to have one unique derived position based on the analysis of a higher quality genome of this isolate that is now available (ELW098). Our analysis identified an additional 4 positions that are unique to genome AGU025. While the coverage of genome AGU020 was deemed too low for inclusion in tree construction, visual inspection of the data revealed it to have at least one read spanning three of the four positions that are unique to AGU025, with identical SNP calls (Table S17). One of these shared positions causes a non-synonymous change in the flavin reductase gene *fre* of the FMN-NADPH reduction pathway (Table S18). An additional position present in only AGU025 affects the *mrcB* gene, which is a penicillin binding protein.

**Figure 5:**
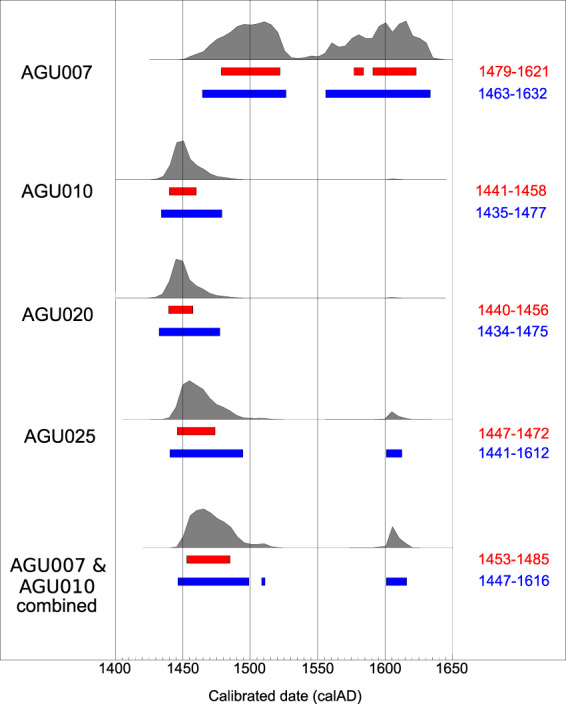
Comparative radiocarbon distribution plots for the individuals with confirmed plague infection. AGU007 (MAMS40889): ^14^C age = 353 ± 18 yrBP, AGU010 (MAMS40326): ^14^C age = 426 ± 19 yrBP, AGU020 (MAMS40328): ^14^C age = 428 ± 19 yrBP, AGU025 (MAMS40329): ^14^C age = 405 ± 18 yrBP. Sigma-1 ranges are shown in red, sigma-2 in blue. The curve for the combined plots of individuals AGU007 and AGU010 is also shown. (created with Oxcal v4.3.2^82^).

Though all burials stem from a common cemetery, it cannot be determined archaeologically whether or not they are from a single mortality event or several events that took place over a series of decades. Radiocarbon dates were thus obtained for all four individuals in whom *Y. pestis* infection was identified (Fig. 5). A conservative estimate based on common overlap of the sigma-2 (95% probability) radiocarbon distributions places all four Lithuanian strains within 12 years of each other, from 1463 CE (oldest age estimate from AGU007) to 1475 CE (youngest age estimate from AGU020). Of note, genome STA001 from Starnberg, Germany has an archaeological date of 1433–1523 CE^22^, which is consistent with our identification of a late 15^th^ century plague outbreak in the Baltics from a more derived lineage. While a contemporaneous co-circulation of the four Lithuanian genomes presented here, which would also include the Ellwangen strain, remains possible based on their overlapping radiocarbon intervals, strain diversity on this scale (4 SNPs) has never been reliably observed in genomic data obtained from any single historical plague outbreak: all published Black Death genomes (1347–1351) are identical^8^, as are four genomes sequenced from a presumed single mortality event in 17^th^ century London^22^. Only a single SNP distinguishes one genotype from the others in the catastrophe cemetery of l’Observance from 1722 Marseille, France^38^ and the 15^th^–17^th^-century Stans mass burial from Switzerland^22^. The identical *Y. pestis* genome from individuals AGU007 and AGU010 thus parsimoniously implies they are contemporaneous. The similarity in radiocarbon distribution between individuals AGU010 and AGU020, the latter of which has at least three additional derived positions, provides support for constraining their age in the mid-15^th^ century. Our phylogeny places genomes AGU007 and AGU010 at the base of a polytomy from which several descendent European lineages arise. As such events in *Y. pestis* evolution have been correlated with large-scale outbreaks^39^, our data provide support for a prolific late 15^th^ century mortality event. As plague outbreaks were, however, frequent in Europe at this time, it is difficult to draw support from the historical literature to add resolution to its time interval. Regardless, it appears this event gave rise to two parallel tracks of persistent European plague: one thus far identified in continental Europe (Germany and Switzerland: LBG, BRA, STN) and another that has thus far been found in the port cities of 17^th^ century London (BED) and 18^th^ century Marseille.

As both London and Marseille were connected to a vast system of trade networks in the Mediterranean and beyond, many candidate plague source locations exist in areas underexplored from the vantage point of ancient DNA. The expansive range of the Ottoman Empire figures naturally into discussions of plague movements and source locations that could have fuelled these later outbreaks: its frequent interactions in the form of either war or trade with Lithuania or its allies in the 15^th^ and 16^th^ centuries^40^ would have provided opportunities for plague introductions from the north such as the AGU007/AGU010 strain identified in this work. Further support for a northern origin comes from the base calls observed at position 4,208,536 (Table S16): although excluded in our phylogeny through application of a 98% partial deletion filter (Fig. 4, Table S16), this position shows the derived state uniquely in genomes AGU007/AGU010, Bedlam (London), and l’Observance (Marseille), thus indicating shared ancestry. Regardless, westerly Mediterranean sources for the inland movement of plague through the Balkans during these periods has received more attention in the historical literature^41^. Of note, the frequency of recorded plague outbreaks in regions under Ottoman control increased abruptly in the late 15^th^ century and persisted thereafter^41^, which is consistent with a regional establishment of the disease, as has been suggested for post-Black Death Europe^8^. A connection between plague and the Ottoman Empire is also consistent with the historical narrative for the Great Plague of Marseille having been introduced via the *Grand*-*Saint-Antoine* merchant ship that collected a variety of trade goods, including supposedly plague-ridden silks, from several Levantine trading destinations days before the disease erupted amongst its crew^42^.

#### *Treponema pallidum*

Phylogenetic relationships of *T. pallidum pallidum* (n = 2), *endemicum* (n = 2), and *pertenue* (n = 27) (Table S19) were determined by calling all variant positions against the Nichols syphilis reference at a minimum coverage threshold of 5-fold and mapping quality of 30 using MultiVCFAnalyzer v0.85 (https://github.com/alexherbig/MultiVCFAnalyzer). Both genomes A12, sampled from a non-human primate^43^, and the 17^th^ century genome from Colonial Mexico^9^ (133) were removed due to low coverage (Table S20). Yaws genomes isolated from non-human primates were included because their phylogenetic placement may inform on directions of host switching. As treponemal genomes are known to recombine^9,44,45^ we sought to identify recombinant regions in our dataset with ClonalFrameML^46^ using a maximum likelihood tree reconstructed in RAxML^37^ with a GTR + GAMMA substitution model for 1000 bootstrap replicates as input. This yielded 54 recombinant regions ranging in size from 3 to 8825 bp, which contained 737 positions that overlapped with, and were subsequently removed from, our list of variants (Table S20). Recombination events specific to AGU007 were not observed. Presence of additional homoplastic positions between yaws and either syphilis or bejel were identified through automated filtering on the SNP table (see supplementary methods). This process revealed an additional 13 positions that were removed for phylogeny construction and molecular dating (Table S21).

**Figure 6:**
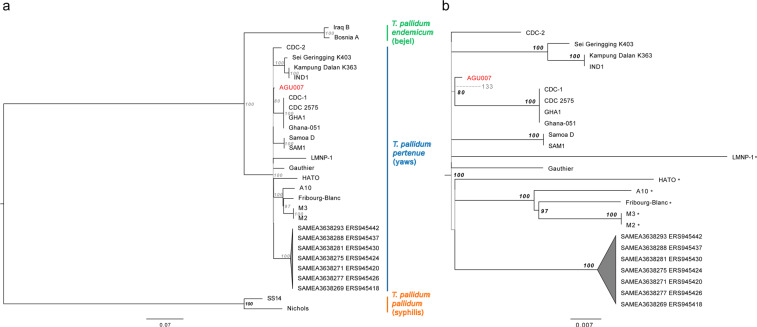
Maximum likelihood phylogenetic tree of *T. pallidum* with 1000 bootstrap replicates generated after removal of 13 homoplastic positions and 54 regions of recombination, followed by deletion of all sites with ambiguous bases. SNP calling was based on a coverage of 5-fold, and the tree was constructed from 929,012 positions of which 1223 were polymorphic. Figure 6a shows a phylogeny of the full treponemal dataset considered here and 6b shows a zoom of the monophyletic clade of *pertenue* strains, where all those isolated from non-human primates are distinguished with an asterisk (*). Branches with statistical support less than 80 are depicted in grey. The scale represents mean substitutions per site given a GTR + GAMMA substitution model. Genome AGU007 is shown in red. The dashed branch and faded text depicts the extrapolated position of ancient genome 133.

The SNP alignment generated after removal of detectable recombinant and homoplastic sites was used as input for phylogeny construction in RAxML^37^ with parameters as described above. All trees consistently yielded high statistical support for discrete grouping of the three treponemal sub-clades of *pallidum* (syphilis, n = 2), *pertenue* (yaws, n = 25), and *endemicum* (bejel, n = 2). Figure 6 shows an estimate of topology based on removal of all sites with missing data. The genomes considered here derive from an extensive polytomy indicative of an expansive event coincident with the emergence of all currently available yaws genotypes. Our ancient genome AGU007 groups consistently with genomes recently typed from human cases in Ghana, West Africa (CDC-1, CDC 2575^47^, and Ghana-051^47^). In addition, AGU007 has a much shorter branch length than its modern counterparts, as expected of ancient genotypes^48^. When all sites are considered, AGU007 differs from the extrapolated yaws MRCA by only five positions (Table S22), two of which are unique to this genome. Both of these private substitutions cause non-synonymous changes, one in the GroES chaperone protein (position 39,267) and another in a methyl-accepting chemotaxis protein (position 523,975) (Table S23), hence their functional significance is worthy of future investigation. While assessment of genome 133 revealed it to have a coverage considered too low for inclusion in tree construction, manual evaluation of its shared SNPs reveals it to possess the derived state for the position shared between AGU007 and the Ghana cluster, thus placing it within this clade. With regard to the positions unique to AGU007, 133 shows the ancestral state for one and no coverage for the other, hence resolution to infer further details on their relationship is currently lacking (Fig. 6; Table S22).

Our identification of a yaws genome in individual AGU007 could provide important clues on disease dissemination and ecology in post-medieval Europe. Given that this individual is suspected to be contemporaneous with AGU010 based on their identical *Y. pestis* genomes, additional resolution for their extrapolated age was sought through merging their radiocarbon data. This resulted in a calibrated radiocarbon date of 1447–1616 CE at a 95% confidence level (sigma-2), though with 83% of the probability density contained in the interval 1448–1498 CE (Fig. 5). This encompasses a time period consistent with the onset of an unknown acute and physically disfiguring disease in Europe.

### Molecular dating of the yaws cluster

Given the small genetic distance between the genome reconstructed from AGU007 and the ancestral node of all yaws considered here, we employed a Bayesian molecular dating approach to formally estimate the time to the most recent common ancestor (tMRCA) of the *Treponema pallidum pertenue* genomes in our dataset. Molecular dating was performed on the same alignment used for phylogenetic inference. Clock-like structure was evaluated using TempEst^49^ and the MEGA maximum likelihood clock test^50^ prior to molecular dating using BEAST (v2.5.2)^51^. The null hypothesis of equal evolutionary rates throughout the tree was rejected.

Due to the range of possible dates for AGU007 based on radiocarbon intervals, we prepared two models in which we sampled the AGU007 tip date from (1) the sigma-2 date range for the radiocarbon date of AGU007 alone (most conservative estimate, 1464–1633 CE; sig2 model) and (2) given their identical *Y. pestis* genomes, the combined sigma-1 date range for AGU007 and AGU010 (least conservative estimate, 1453–1485 CE; sig1 model). The clock rate was not meaningfully impacted by differences in tip dating ranges for AGU007, as both yielded mean values well within the 95% highest posterior density (HPD) intervals for each model (Figure S8). The mean dates for the tMRCA for the yaws cluster included here differ by only 169 years between the Sig1 and Sig2 models (Table 3). The 95% HPD intervals for this parameter also overlapped substantially and contained the mean value of the alternative model (Figure S8). Together these results suggest an emergence of this cluster within the last millennium.

**Table 3:** Selected parameter estimates from BEAST dating.

Model	Clock Rate (95% HPD)	Yaws tMRCA (95% HPD; YBP)	AGU Tip Date (95% HPD)
Sig1	4.6E-8 (2.4E-8, 6.9E-8)	865 (571, 1235)	1469 CE (1455, 1485)
Sig2	5.8E-8 (2.7E-8, 8.8E-8)	696 (424, 1043)	1561 CE (1477, 1633)

## Discussion

Our recovery of *T. pallidum pertenue* in archaeological human remains from post-medieval Lithuania invites an exploration of the historical context surrounding treponemal infections during this period. In the late 15^th^ century, historical accounts begin to accumulate in Europe^52^ and the Middle East^53^ that describe a physically disfiguring disease seemingly unknown to medicine at the time. Early documents surfaced in Spain with Ruy Díaz de Isla’s description of “bubas”, though more widely accepted is Gonzalo Fernández Oviedo y Valdés’s description of a malady that affected the mercenary armies of Charles VIII of France that laid siege to Naples in early 1495^52^. By mid-spring of that year the armies began retreating back to their homelands, though a new, highly contagious, disfiguring and rapidly progressing disease accompanied their military victory. In subsequent decades the illness took on many names such as the “French pox” or the “Pox of Naples”, all of which were more reflective of political and social tensions of the era than biological truths^54^. Today these accounts are widely assumed to be the first documented descriptions of the treponemal disease known as syphilis.

Theories on its origin began to surface shortly after its first classification as a distinct condition, and fervent controversy over the varying hypotheses persists to this day. Most discussants favour the Columbian theory that assumes acquisition from New World populations by Columbus’ crew on their first trans-Atlantic voyage^52^, with its rapid transmission in Europe facilitated through cultures of sexual activity^55^. Genetic data from both human and non-human primate treponemal infections are interpreted as providing support for the theory, and have led to the proposal of a prehistoric emergence of the treponemal disease cluster in the form of yaws in Africa^56^. The spirochete is then thought to have followed modern humans as an “heirloom” disease in their Pleistocene dispersal across the globe, where it adapted locally into the endemic form bejel (*Treponema pallidum endemicum*) in the Middle East and several hypothetical sub-types of yaws (*Treponema pallidum pertenue*) in both the New and Old World. Introduction of New World yaws to 15^th^ century Europe is then thought to have provided the ecological context for the emergence of the sexually transmitted variety, syphilis (*Treponema pallidum pallidum*)^52,57^. Phylogenetic relationships revealed from genome-level analyses^9,43,44^ show cladistic separation of the three treponemal pathogens, and while they share a common origin, there is no support for yaws as the ancestor. Dispersal of yaws in both humans and non-human primates, however, is still thought compatible with it being an heirloom disease^58^: this would push emergence of the pathogenic treponemal cluster far back into the Pleistocene, leaving open multiple models compatible with an early evolution in the Americas^58^.

Though the theory of a New World origin is almost as old as historical documentation of the disease itself^59^, it is not without critics. A growing body of evidence from the meticulous recording of skeletal lesions considered pathognomonic for syphilis identified in pre-Columbian Europe challenges popular assumptions on its origin. Under this model, syphilis may well have been present in Europe for centuries before New World contact, though may have been obscured by alternative nomenclature such as “leprosy”, a singular term thought to have described a variety of disfiguring conditions in antiquity. Syphilis could then have been among the many diseases brought to the New World via European contact. Both theories are fuelled by analyses of skeletal material from New^60^ and Old World contexts^61–63^, with the latter questioned by the Columbianists based on scepticism about diagnostic resolution for syphilis pathology in archaeological bone or the influence of marine reservoir effects on radiocarbon dates^57,64^. Others contend that these pre-Columbian forms could result from related treponemal infections, which were later replaced by syphilis in the late 15^th^ century^65^. In contrast, sparser attention is given to the “alternative” theory^66^, which also assumes a treponemal emergence in sub-Saharan Africa at some unspecified time in the past, though here either syphilis or its immediate predecessor became introduced to Europe via increased trade contacts with West Africa in the late 15^th^ century. This less popular scenario is thus entirely independent of New World involvement, and emergence of the disease in Europe would have been merely correlative with Columbus’ travels^67^.

Importantly, all three hypotheses operate on the assumption that the new disease of late 15^th^ century Europe was unequivocally an intense epidemic of the sexually transmitted *Treponema pallidum pallidum*. Its rapid onslaught and perceived decrease in virulence within half a century is frequently cited as a hallmark example of an emergent disease within a naïve host population^68^, wherein either pathogen or host rapidly adapt to achieve a closer approximation of a symbiotic relationship^52,56,69^. Here we propose a hypothesis that is to our knowledge novel and requires fewer assumptions to explain the epidemiological trends – that yaws be considered an important contributor to the late 15^th^ century European pandemic. Yaws, and for that matter the other currently recognised treponemal diseases bejel and pinta, have not figured into discussions and theories of treponemal ecology in medieval Europe because their modern distribution reveals them to be restricted to warmer climates. Regions of sub-Saharan Africa, Southeast Asia, and Oceania are current targets for yaws eradication efforts, where the disease is invariably linked to rural areas lacking adequate resources to sustain global hygienic standards^70–72^. Our unexpected finding of yaws in a European individual from the Baltics, where skeletal evidence of pathognomonic treponemal pathology is purportedly absent prior to the 16^th^ century^67^, reveals that at least the past form of the disease was indeed infectious under climatic conditions that differ from its current niche. Its phylogenetic position in close proximity to the radial divergence of all known members of the yaws cluster permits the establishment of a temporal dimension for its evolution, further supported by our dating estimates, and demonstrates the disease, as it is currently defined, to be far younger than previously assumed^56^ – with an inferred emergence in the 12^th^–14^th^ century. This recent emergence would imply that any older material presenting treponemal pathology results from strains that are not defined by the current yaws cluster.

Although the lack of a clear ancestor-descendent relationship with any typed lineage prevents resolution on directions of transmission between humans and non-human primates, the close relationship of our ancient genome to extant West African lineages, coupled with the diversity observed in other modern African yaws genomes, is consistent with its emergence in this region. As noted by the alternative theory, increased European presence in West Africa in the mid- to late-15^th^ century would have provided a means for intercontinental disease movement^66^. The lure of West African gold motivated the Portuguese establishment of Elmina (modern Ghana) in 1478, though its activities quickly expanded beyond trade of minerals to include importation of African people to Europe as slaves. By the mid-sixteenth century, an estimated 10% of Lisbon’s population was of African descent^73^. Introduction of a highly contagious skin infection such as yaws during this period could have easily led to its presence in the 20,000 strong 1494 mercenary army of Charles VIII, whose contingent was assembled from a wide recruitment of men across Europe^74^.

Cutaneous lesions from yaws infections can occur anywhere in the body, and usually take the form of raised chancres similar in shape to a wild raspberry. Such pathology is consistent with reports of “welts the size of acorns” that described early symptoms of the 15^th^ century disease^75^. Skeletal involvement in yaws is common, and can be manifested as dactylitis and periostitis of long bones. Later stages of the disease, if left untreated, can result in tibial bowing, caries sicca on the skull and gangosa of the nasal bones and maxilla, hence comprising a spectrum of pathology that can be indistinguishable from advanced syphilis in archaeological^54,76^ and even modern clinical contexts^71^. Recovery with subsequent immunity is common today, and contributes to the disease’s reputation as an illness of children in remote and underserviced areas. Immunity amongst survivors of the historical condition could have been a driving mechanism behind the perceived reduction in virulence of the causative agent for the post-Columbian European outbreak, and would also be consistent with its low-level persistence into the 19^th^ century in Europe, potentially in a variety of rare forms such as sibbens of Scotland, button scurvy of Ireland, and radesyge of Norway^77^, all of which receded with changing standards of personal hygiene. Though the skeletal manifestation in the Lithuanian woman investigated here is consistent with, though not specific to, endemic (or non-venereal) treponemal pathology, our identification of ancient yaws DNA in one of her teeth is confirmatory. As yaws infection undergoes a short period of haematogenous involvement in advance of prolonged infection, we regard the presence of treponemal DNA in the dental pulp chamber as stemming from increased vascular permeability associated with the acute septic progression of plague, coincident with an underlying and pre-existing active infection of yaws. This proposed causal relationship implies merit in deeper non-targeted pathogen surveys of skeletal collections from known periods of high mortality associated with the many examples of plague or other septic diseases in our history. The further support we demonstrate for plague’s endemic status in post-medieval Europe and its continued potential for large-scale outbreaks beyond the Black Death would have provided a wealth of opportunity for co-morbidity and sepsis for a variety of infections.

Earlier pessimism regarding the inaccessibility of treponemal diseases in archaeological material were made on the assumption of the spirochete’s fragile structure, its limited *in vivo* detection in animal bone under controlled laboratory infection trials, and simple lack of success in PCR amplifications from ancient bone of presumed treponemal sufferers^78,79^. As the roster of archaeological samples showing molecular preservation of these diseases increases^9,80,81^, and their detection is further enhanced by analytical advances, the resolution of enigmas related to the origins of the full treponemal cluster, inclusive of its other members syphilis, bejel, and pinta, are within reach. Such feats are likely to be realised in the coming years as the burgeoning field of palaeopathogenomics continues to mature and move beyond previously established boundaries.

## Methods

Additional methods can be found in our supplementary file.

## Supplementary information

Supplementary information.

Supplementary information 2.

## Data Availability

Raw data from the above analyses can be found under ENA accession number PRJEB37508.
