# Supplementary material for: A treponemal genome from an historic plague victim supports a recent emergence of yaws and its presence in 15th century Europe
Source: Sci Rep. 2020 Jun 11;10:9499. doi: 10.1038/s41598-020-66012-x (PMC7290034; doi:10.1038/s41598-020-66012-x)
Supplement: Supplementary file 2 — Supplementary information 2. [file 41598_2020_66012_MOESM2_ESM.pdf]

Supplementary Information

A treponemal genome from an historic plague victim supports a recent emergence of yaws and its presence in 15th century Europe

Karen Giffin[1]*, Aditya Kumar Lankapalli[1]*, Susanna Sabin[1], Maria A. Spyrou[1], Cosimo Posth[1], Justina Kozakaitė[2], Ronny Friedrich[3], Žydrūnė Miliauskienė[2], Rimantas Jankauskas[2], Alexander Herbig[1], and Kirsten I. Bos[1]

[1]Max Planck Institute for the Science of Human History, Jena, Germany
[2] Vilnius University, Vilnius, Lithuania
[3] Curt-Engelhorn-Zentrum Archäometrie, Mannheim, Germany

DNA extraction and qPCR Screening

Sampling, DNA extraction, qPCR reaction preparation, library preparation and indexing reaction preparation were performed in the ancient DNA clean lab facilities of the Max Planck Institute for the Science of Human History. All amplifications were carried out in the modern DNA lab facilities of this institution.

One tooth from each of 26 individuals was sectioned, and the pulp chamber was drilled using a Dremel tool at low setting affixed with a grout-cutting bit. DNA was extracted from ca. 50 mg of powder using a protocol designed to increase the recovery of short fragments, which are typical of ancient DNA[1]. The extraction protocol was modified as per Spyrou et al., 2019. Two extraction blanks were analyzed along with each set of 10 samples, the first as a reagent blank, and the second as a process blank. An inhibition test[2] was performed to rule out the presence of PCR-inhibiting components by spiking 2 µL of sample extract into Illumina library qPCR reactions, with known amounts of standard template molecules, to record deviations from the anticipated amplification behaviour. No inhibition was detected via this method. Reaction chemistry for the *pla* qPCR assay for detection of *Y. pestis* PCP1 plasmid DNA was performed without deviation[3]. Results of the *pla* assay can be found in Figures S1 and S2, and Table S1.

Library preparation and shotgun sequencing

i) Library preparation and sequencing

Double-stranded Illumina libraries were prepared for all 26 individuals and their associated negative controls using 10 µL of DNA extract following established protocols[4]. Additionally, for the 6 putative positive plague samples, double-stranded libraries were prepared using 40 µL of DNA extract to increase template. Prior to adaptor ligation, each enriched library was treated with uracil-DNA-glycosylase (UDG) and Endonuclease VIII (New England Biolabs) to repair damaged DNA bases[5,6]. Two library blanks were analyzed along with each set of samples, the first a reagent blank, and the second a process blank. Quantification of libraries, indexing, and quantifications of the indexed libraries were performed as per Spyrou et al., 2019. The libraries of the UDG and nonUDG samples and blanks were amplified to individual concentrations of approximately $1.3*10^{13}$ DNA copies/uL using Herculase II Fusion DNA Polymerase (Agilent), purified as per Spyrou et al. (2019). Samples and blanks were pooled separately to total concentrations of approximately 10nM for Illumina shotgun sequencing. Concentrations were measured on a TapeStation 4200 (Agilent). Aliquots of the amplified UDG and nonUDG libraries for the 6 putative plague positives and their associated blanks were further amplified for capture to a concentration of 200-400 ng/µL as per Spyrou et al. (2019). Concentrations of capture libraries were measured on a Nanodrop 8000 (Thermo Scientific). In-solution *Y. pestis* capture was

performed as previously described[7]. Samples were captured in individual wells, but blanks were pooled and captured together separately from the samples.

Shotgun libraries and capture products were sequenced to a depth of approximately 10 million reads on an Illumina HiSeq4000 using a 50 bp paired-end kit. Screening and captured blanks were sequenced to a depth of approximately 2 million on an Illumina NextSeq500 on either a 75 bp paired-end mid-output kit or on a 75bp single-read kit, except for the *T. pallidum* capture blanks for the first tooth from AGU007, which were sequenced to a depth of 2 million reads on an Illumina HiSeq4000 using a 75bp single-read kit.

ii) Illumina read processing

Sequenced shotgun libraries and blanks were demultiplexed and mapped to the human genome (hg19) in EAGER v1.92 to evaluate aDNA preservation[8]. In EAGER, reads had the adapters clipped, and paired reads were merged using AdapterRemoval v2.2[9]. Reads were filtered for a length of ≥ 30 bp and a minimum base quality of 20. Mapping was performed by BWA version 0.7.12 with a seed length (-*l*) of 32 and a mapping stringency setting of 0.01 (-*n*). We removed read alignments with a mapping quality (-*q*) below 20 using SAMtools (http://samtools.sourceforge.net/). Duplicates were removed with MarkDuplicates (http://broadinstitute.github.io/picard/) and mapDamage v2.0 was used to examine DNA damage[10]. The 26 samples had 373 – 1,090,755 DNA fragments mapping to hg19 after quality filtering and duplicate removal, and % endogenous DNA ranging from 0.01 to 32.61 (Table S2). Negative controls showed between 90 and 24,782 fragments mapping to hg19 after quality filtering (Table S2).

Shotgun libraries for samples and blanks, both UDG and nonUDG, were mapped in EAGER against the CO92 reference genome for *Y. pestis* (NC_003143.1) as a verification of the qPCR screening results[11]. EAGER was applied as described above, however, the BWA mapping parameters were -*l* = 16, -*n* = 0.01 and read alignments with a mapping quality below 37 were removed. Results are presented in Table S3.

iii) qPCR re-evaluation

On account of the unexpectedly high number of mapping *Y. pestis* fragments in individual AGU007, re-examination of the qPCR assay results revealed that AGU007 had a signal that had been initially dismissed. AGU007's amplification curve indicated a quantity below the lowest standard of 0.2 copies/µL (Figure S1, Table S1), and the melting peak signal was also very low as compared to the amplification standards and the other samples (Figure S2).

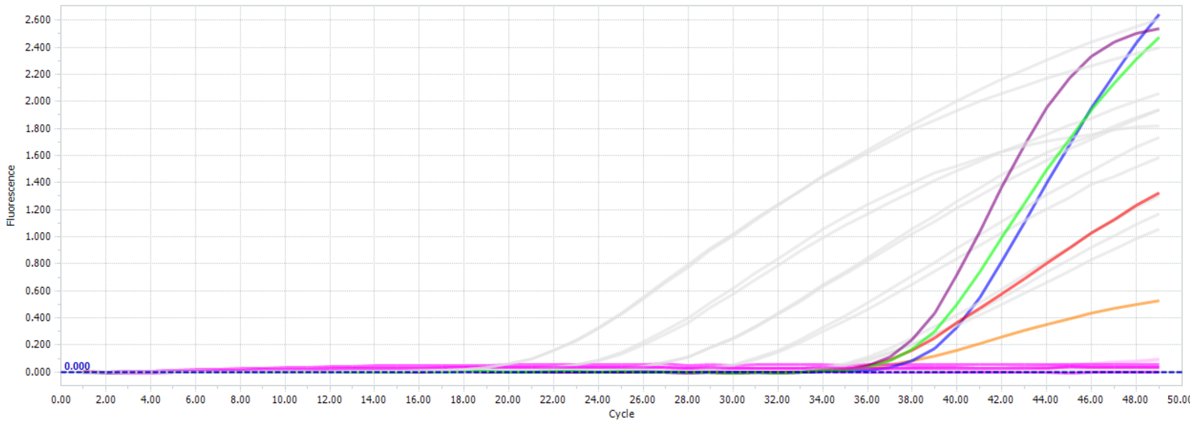

Figure S1 – Amplification curves of pla Assay. Curves in grey are *pla* standards. Purple, green, blue, red and orange curves are AGU010, AGU009, AGU013, AGU003 and AGU007, respectively. Extraction and qPCR blanks are shown in pink.

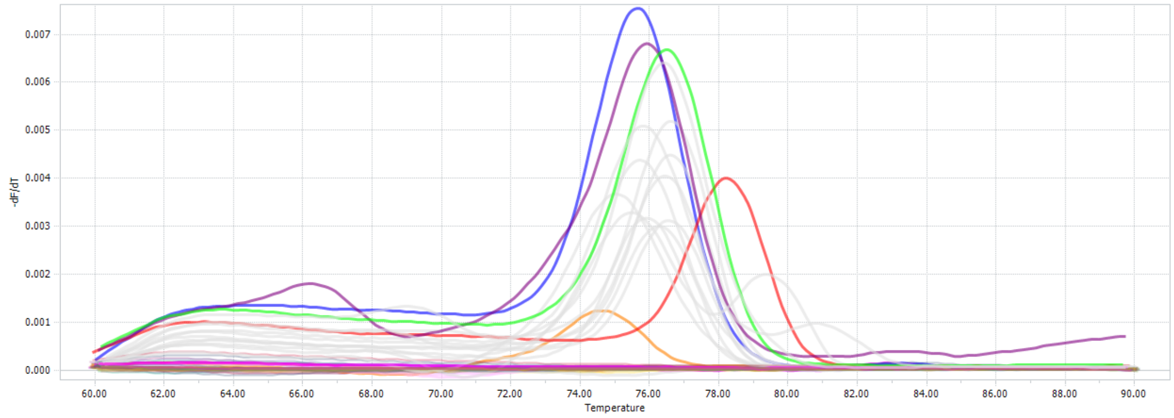

Figure S2 – Melting peaks of pla Assay. Curves in grey are *pla* standards. Purple, green, blue, red and orange curves are AGU010, AGU009, AGU013, AGU003 and AGU007, respectively. Extraction and qPCR blanks are shown in pink.

A UDG library from 40 µL of extract was prepared for AGU007 and was shotgun sequenced on an Illumina HiSeq 4000 with a 75bp single end kit. Mapping to hg19 and *Y. pestis* was carried out as previously described, though with more stringent parameters (-*l* 32, -*n* 0.1, -*q* 37) for the *Y. pestis* mapping (Table S3).

Pathogen Screening in HOPS

Libraries were screened for the presence of ancient pathogens using the MEGAN Alignment Tool (MALT) as part of the Heuristic Operations for Pathogen Screening (HOPS) pipeline[12,13]. The MALT database used for screening was constructed from a custom RefSeq Genome set in November 2017 that contained bacteria, viruses, and eukaryotes (ftp://ftp.ncbi.nlm.nih.gov/genomes/refseq/). *Y. pestis* signals were observed in the nonUDG library of AGU007, and in both the UDG-treated and untreated libraries for

AGU010, AGU020 and AGU025. Contrary to the qPCR assay, no *Y. pestis* signals were observed in either the UDG-treated or untreated libraries for AGU003, AGU009, and AGU013, indicating these were likely false positives.

Aside from *Y. pestis*, numerous reads were assigned to other pathogenic taxa, including oral microorganisms (Figure S3). Verification of these signals would require targeted enrichment for species and strains of the relevant taxa, and was not pursued here.

In addition to *Y. pestis*, the UDG library for AGU007 also demonstrated a signal for *Treponema pallidum* that had not been observed in the initial screening of the nonUDG library. Closer examination of the MALT data of the nonUDG library disclosed the presence of 7 fragments matching to *T. pallidum* and 11 fragments matching to the *Treponema* genus (Figure S3). This was not reported as positive identification by HOPS since the level of damage in the reads precluded proper evaluation of taxon assignment.

**Organism**	**AGU007.A**		**AGU010.A**		**AGU020.A**		**AGU025.A**	
	**nonUDG**	**UDG**	**nonUDG**	**UDG**	**nonUDG**	**UDG**	**nonUDG**	**UDG**
***Yersinia pseudotuberculosis* complex**	90	83	29	30	36	38	30	26
Yersinia pestis	19	17	9	10	8	8	9	7
Tannerella forsythia							1055	1602
Treponema pallidum pallidum	7	12						
Treponema	11	21						18
Streptococcus anginosus							4	
Streptcoccus agalactiae							2	
Streptococcus							15	
Schistosoma mansoni							2	
Salmonella enterica enterica								9
Porphyromonas gingivalis							118	159
Parvimonas micra							160	226
Neisseria						18		11
***Mycobacterium tuberculosis* complex**					5			
Mycobacterium leprae					4			
Mycobacterium intracellulare	26							
Escherichia coli			260	228				
Enterobius vermicularis		115		13				
***Clostridium botulinum* BKT015925**							5	
**Borreliaceae**								2

Figure S3 – Pathogen screening data produced in HOPS from the putative *Y. pestis* positive screening libraries. Numbers in each cell correspond to assigned reads. Cell fill colour indicates degree of taxon assignment evaluation: gold pass the set edit distance filter, green pass this filter and also have terminal C to T damage, and blue shows that reads with terminal C to T damage pass the edit distance filter. Grey indicates no positive taxon assignment evaluation in HOPS.

Both the untreated and UDG-treated libraries were mapped in EAGER against the Nichols syphilis reference genome (NC_021490.2) using the same programs and parameters as previously described for mapping to CO92[14]. The untreated library was found to contain 27 mapping fragments after duplicate removal and quality filtering and the UDG-treated library was determined to have 36 mapping fragments following duplicate removal and quality

filtering (Table S11). The other 25 AGU samples had mapping fragment numbers ranging from 0-8 after duplicate removal and quality filtering. Screening blanks were also mapped in EAGER using screening library parameters and programs, and were found to have 0-3 fragments mapping to Nichols (NC_021490.2) after duplicate removal and quality filtering, consistent with the other samples found to be negative via screening.

For targeted enrichment of *Treponema pallidum*, DNA probes were designed on the basis of *Treponema pallidum* subsp. *pallidum* strains Nichols (NC_000919.1), SS14 (NC_021508.1), Sea 81-4 (NZ_CP003679.1), Mexico A (NC_018722.1), *T. pallidum* subsp. *endemicum* strain Bosnia A (NZ_CP007548.1), and *T. pallidum* subsp. *pertenue* strain Fribourg-Blanc (NC_021179.1). The probes were designed with a 1bp tiling and a length of 52 bp with an additional 8bp linker sequence (CACTGCGG) as described in Fu et al. (2013)[15]. Duplicated probes and probes with low sequence complexity were removed. This resulted in 1,125,985 unique probe sequences. This probe set was spread on two Agilent one-million feature SureSelect DNA Capture Arrays. The capacity of the two arrays was filled by randomly duplicating probes from the probe set. The arrays were turned into an in-solution DNA capture library as described elsewhere[15].

The treated and untreated libraries for AGU007 and its associated blanks were then in-solution captured for whole genomic *Treponema pallidum* DNA using the capture library described above. The *T. pallidum*-captured untreated and UDG-treated libraries were captured in individual wells and the associated blanks were pooled and captured separately from the samples.

Mapping of captured products for *Y. pestis* and *T. pallidum* (first tooth)

Reads from *Y. pestis*-captured samples were mapped in EAGER against the CO92 reference genome (NC_003143.1)[11] with stringent BWA mapping parameters (-*l* 32, -*n* 0.1, -*q* 37). Samples had 119 – 156,123 fragments mapping to CO92 after quality filtering and duplicate removal, with mean genomic coverages ranging from 0 to 1.98-fold (Table S4 and S5). Untreated libraries showed expected damage patterns (Figure S4). Samples AGU003, AGU009 and AGU013 had mean genomic coverages of 0 – 0.01-fold following capture, supporting the negative MALT/HOPS results. Samples AGU010, AGU020 and AGU025 had low mean genomic coverages ranging from 0.28 to 0.77-fold. Captured blanks had between 5 and 90 mapping fragments, with duplication (cluster) factors ranging from 2.3 to 2645.7, indicating that complexity of the library had been adequately explored. These numbers are consistent with previously reported *Y. pestis* capture blank data in Bos et al. 2016.

The *T. pallidum*-captured untreated and UDG-treated libraries for AGU007 were sequenced on a HiSeq4000 to a depth of approximately 40 million reads with a 75 bp paired-end kit. The samples were mapped in EAGER to the Nichols genome (NC_021490.2) with the same

parameters as the *Y. pestis* mapping. The libraries had 31,882 and 126,552 mapping fragments, respectively, following duplicate removal and quality filtering (Table S12). The mean coverages of the untreated and UDG-treated libraries were 1.78X and 6.67X, respectively, with the untreated library displaying the expected aDNA damage pattern (Figure S5). The UDG-treated library covered 96.1% of the genome at 1X and 76.1% at 5X. Captured blanks, sequenced on a HiSeq4000 with a 75bp single read kit, had 4 or fewer reads mapping to the Nichols genome after quality filtering.

Processing and analysis of Second Set of Teeth from AGU

Further data analysis awaited the arrival of the second set of teeth: one each from individuals AGU007, AGU010, AGU020 and AGU025. Following arrival, sampling, DNA extraction, library preparation, indexing, amplification, and *Y. pestis* and *T. pallidum* capture protocols were followed as previously described. No qPCR screening was performed for these samples. Remaining aliquots of unindexed AGU010 and AGU020 UDG-treated libraries from the first tooth were indexed and captured as part of the same batch following the same processes. Untreated and UDG-treated screening libraries were sequenced on separate runs on a HiSeq4000 using single-end 75 bp kits. *Y. pestis*-captured libraries were paired end sequenced to a depth of 20 million reads on a NextSeq500 using a 75bp mid output kit and *T. pallidum*-captured libraries were sequenced to a depth of approximately 40 million on a HiSeq4000 using a 75 bp paired-end kit. All negative controls were sequenced to a depth of approximately 2 million on separate NextSeq500 runs using 75 bp mid-output paired end kits.

Mapping of the untreated and UDG-treated libraries of the tooth samples to hg19 was performed following the same procedures described previously. Shotgun sequencing yielded endogenous DNA contents of 0.07-59%, which corresponded to 6,381 – 6,986,467 mapping fragments after duplicate removal and quality filtering (Table S6). Both AGU007 and AGU010 had better human DNA preservation in the second tooth, but the opposite was observed for AGU020 and 025. Negative controls showed between 683 and 6,886 fragments mapping to hg19 after quality filtering. Mapping of the untreated and UDG-treated shotgun libraries to the *Y. pestis* CO92 genome was also performed, yielding 22 – 6,394 mapping fragments following duplicate removal and quality filtering (Table S7). UDG-treated shotgun libraries were mapped using more stringent parameters than untreated shotgun libraries (-*l* 32, -*n* 0.1 and -*q* 37, and -*l* 16, -*n* 0.01 and -*q* 37, respectively). Negative controls had 11 or fewer fragments mapping after quality filtering.

Mapping of shotgun libraries from the second set of teeth against the Nichols genome (NC_021490.2) yielded 1 – 181 fragments following duplicate removal and quality filtering (Table S13). Mapping parameters used were the same as for the shotgun library mapping to

Y. pestis. Blanks showed no indication of the presence of *T. pallidum* DNA after quality filtering.

Shotgun libraries for these second teeth were all screened using MALT/HOPS. As expected both *T. pallidum* and *Y. pestis* were detected in the UDG-treated and untreated libraries for AGU007. *Y. pestis* was detected in AGU010, AGU020 and AGU025, though for AGU020 it was detected in only the UDG-treated library, suggesting that *Y. pestis* DNA levels were minimal.

In-solution *Y. pestis* capture was performed on all libraries and their associated blanks using previously described methods[7]. Libraries for AGU007 and its blanks were also captured for *T. pallidum* as described above. The captures for both *Y. pestis* and *T. pallidum* were initially sequenced together on an Illumina HiSeq4000. The *Y. pestis* captures were subsequently re-sequenced separately on an Illumina NextSeq500.

Mapping of the *Y. pestis*-captured samples and blanks in EAGER against CO92 yielded 3978 – 2,344,695 mapping fragments and mean coverages of 0.04X to 38.01X after duplicate removal and quality filtering (Table S8). Captured nonUDG libraries showed the expected ancient DNA damage pattern (Figure S4). AGU020 had the lowest coverage, 0.04X and 0.09X for its untreated and UDG-treated libraries, respectively. By contrast, the UDG treated AGU007 had a mean coverage of 38.01-fold with 93.7% of the genome covered at 5-fold. AGU010 had a mean coverage of 20.76-fold with 92.0% of the genome covered at 5-fold. AGU025.B had a mean coverage of 14.36-fold with 92.1% of the genome covered at 5-fold. For maximum coverage, all available sequencing data for the first tooth of AGU020 from the UDG library aliquots of both captures were merged. This resulted in a coverage of 1.68-fold. *Y. pestis*-captured blanks had 3-24 fragments mapping to *Y. pestis* after duplicate removal and quality filtering, with cluster factors between 1 and 1092.

Verification of the presence of *Y. pestis* plasmids pMT1 and pCD1 was also performed. Mapping in EAGER using previous stringent parameters yielded coverages for the pMT1 from 4.02 to 63.75-fold, with 6284 to 79,105 fragments mapping after quality filtering. As expected, the lowest values were observed for AGU020 and the highest for AGU007 Table S9). No more than 4 uniquely mapping fragments were observed in the captured negative controls. Mapping to pCD1 displayed similar results with the lowest values found in AGU020, with 6983 unique mapping fragments and 6.08-fold coverage, and the highest in AGU007, with 68,941 uniquely mapping fragments covering the pCD1 at 80.90-fold (Table S10). No uniquely mapping fragments were found in the negative controls.

The *T. pallidum*-captured libraries of AGU007 and its associated blanks were mapped in EAGER against Nichols (NC_021490.2) using the same parameters and programs as the *Y. pestis* captures. AGU007 had 146,398 – 330,505 fragments mapping after duplicate removal

and quality filtering, mean coverages of 9.3 to 21.3-fold and 92.1% to 97.6% of the genome covered at 5-fold (Table S14). The nonUDG library showed the expected ancient DNA damage pattern (Figure S5). One mapping fragment was detected in three of the captured blanks after quality filtering. Each mapping fragment was analyzed using BLASTn and found to map to regions conserved in multiple bacterial taxa. None of the top 10 matches were to *T. pallidum.*

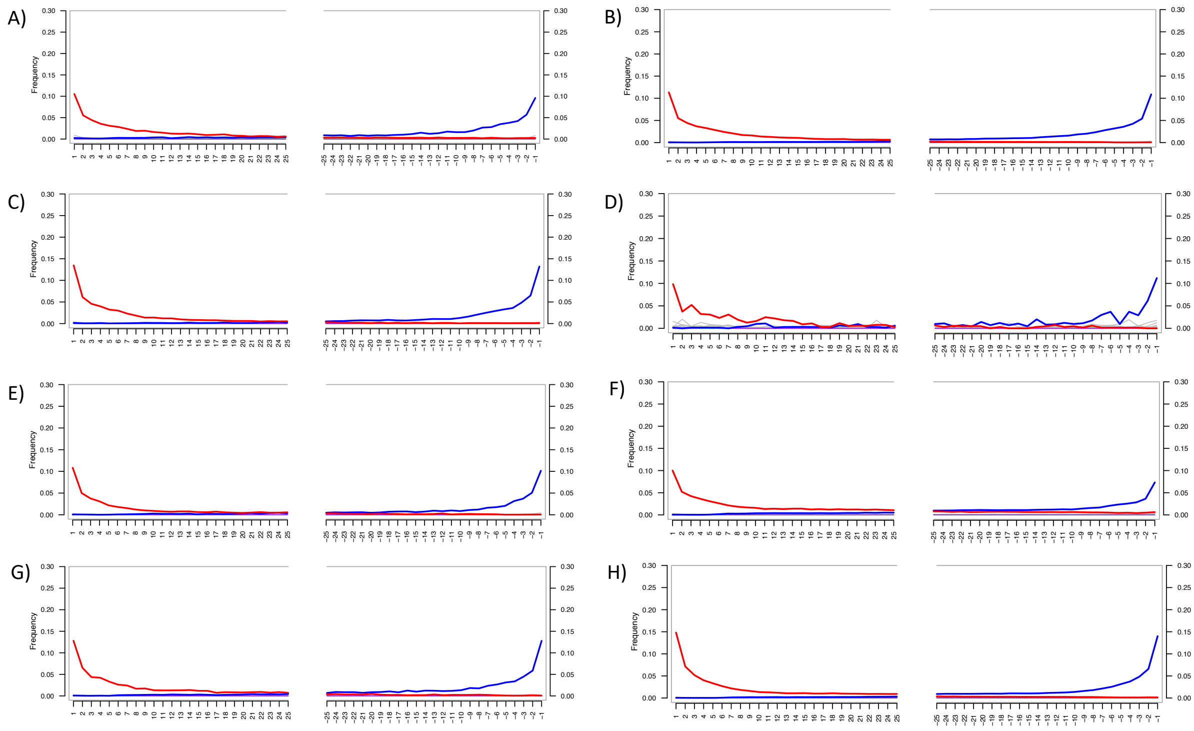

Figure S4 – *Y. pestis* DNA damage plots, generated by Mapdamage 2.0 in the EAGER pipeline, of captured libraries of A) AGU010 tooth 1, B) AGU010 tooth 2, C) AGU020 tooth 1, D) AGU020 tooth 2, E) AGU025 tooth 1, F) AGU025 tooth 2, G) AGU007 tooth 1 and H) AGU007 tooth 2.

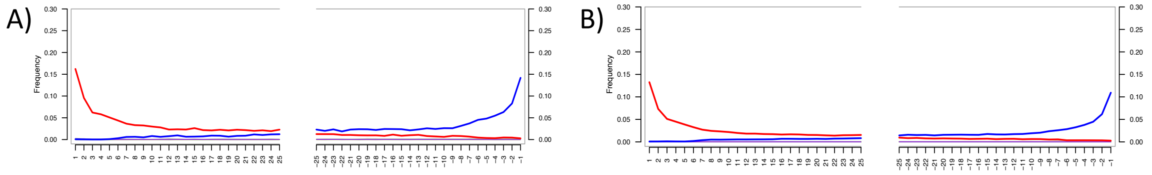

Figure S5 – *T. pallidum* DNA damage plots for captured libraries of A) AGU007 tooth 1 and B) AGU007 tooth 2.

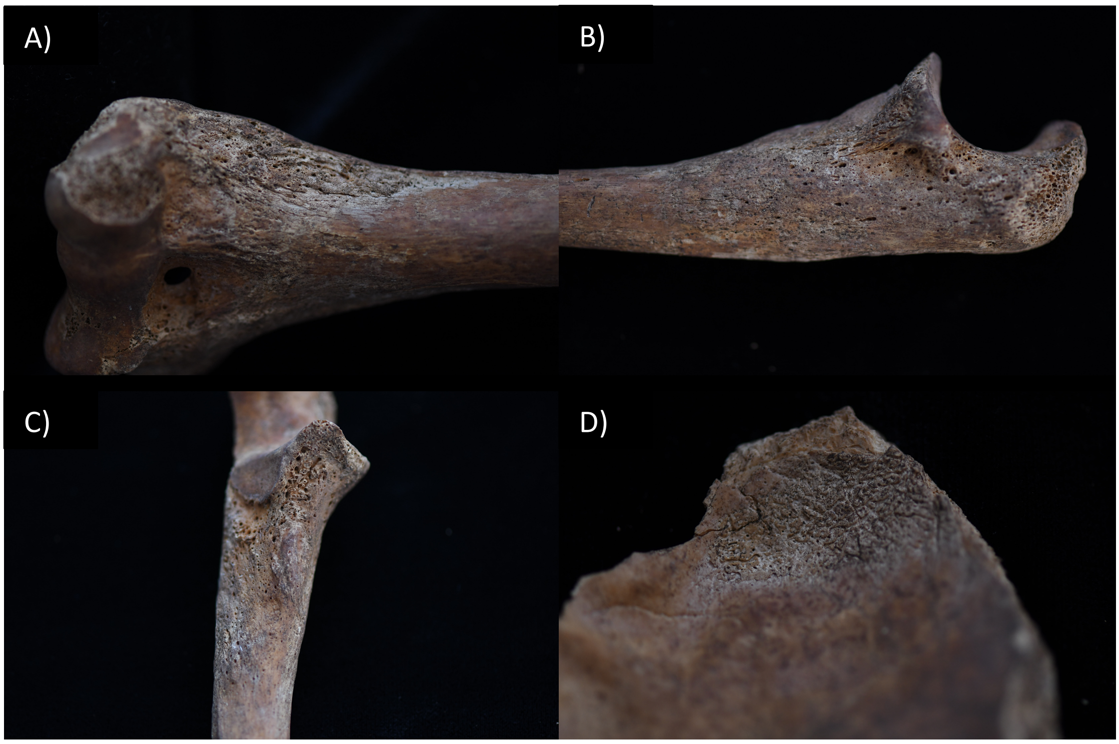

Figure S6 - Paleopathological Features of the skeleton of AGU007, A) right humerus, B) lateral view of right ulna, C) anterior view of the olecranon process of the right ulna, D) parietal bone (photographs courtesy of Justina Kozakaitė).

Phylogenetic Analysis of *Yersinia pestis*

Phylogenetic assessment was made following methods described in the main manuscript with a genome dataset defined in Table S15. *Y. pseudotuberculosis* was used as the outgroup. A full phylogeny is presented in figure S7, and a zoomed in phylogeny of the post-Black Death lineages only is shown in Figure 4.

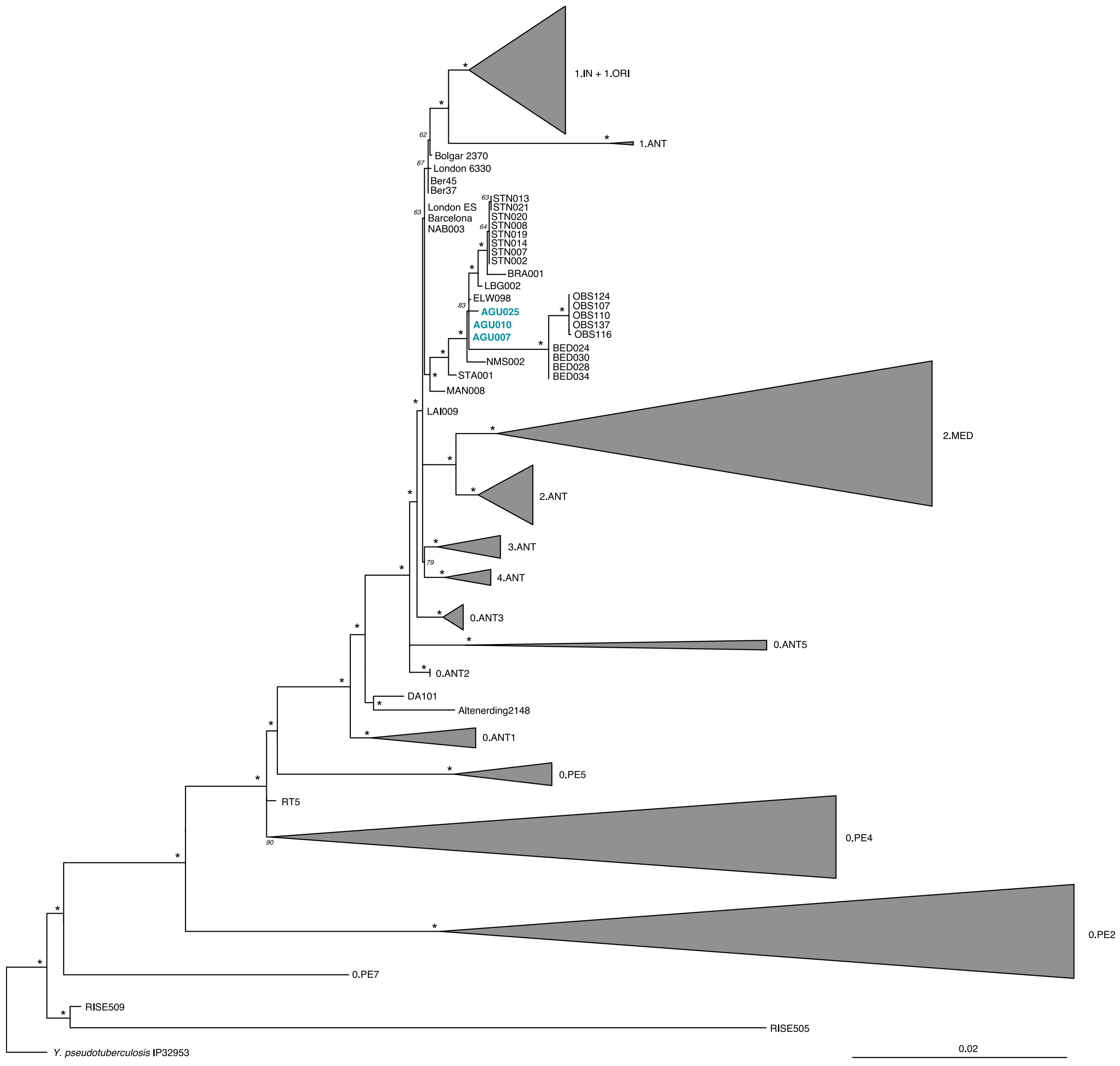

Figure S7: Maximum likelihood tree of post-Black Death genomes of *Y. pestis*. Constructed from 275 genomes with the Generalised Time Reversible (GTR) model, 1000 bootstrap replicates, and a 98% partial deletion filter (considering 5801 SNPs). Bootstrap values of 95 or greater are indicated with an asterisk (*). Scale denotes substitutions per site.

Phylogenetic Analysis of *Treponema pallidum pertenue*

Sequencing reads for 70 samples from Arora *et al.*, 2016[16], 8 samples from Marks *et al.* 2018[17], and 8 samples from Knauf *et al.*, 2017[18] were analysed along with simulated reads generated from complete *T. pallidum* genomes downloaded from NCBI of 1st April,2019 (Table S19). Each simulated read is 100bp long with 1bp tiling to the successive read. Adapters of sequenced reads were trimmed and poor-quality reads were filtered. Overlapping reads were merged and processed in EAGER version 1.92.58[19]. For AGU007,

raw reads from the first and second rounds of capture were filtered for sequencing quality independently. The resulting *fastq* reads were combined to form the raw data for the AGU007 merged dataset. These reads were mapped against reference genome *Treponema pallidum* subsp. *pallidum* Nichols (NC_021490.2) with mapping quality of 37, seed length of 32 with 0.1 fraction of missing alignment against the reference. Duplicated reads with the same 5' end, but of lower quality were excluded using MarkDuplicates (http://broadinstitute.github.io/picard/). Variants were called using GATK[20]. Using a threshold of five-fold mean coverage, 33 genomes covering 90 percent of the reference Nichols genome covered were of sufficient quality to be carried forward in our analysis, and their vcf files were combined and compared for downstream analysis. Variant positions were identified from a set of 2 *pallidum* genomes, 2 *endemicum* genomes and 25 *pertenue* genomes (Table S20) using MultiVCFAnalyser (https://github.com/alexherbig/MultiVCFAnalyzer). SNP calling was based on a minimum coverage of five reads with a minimal mapping quality of 30 and at least 90 percent of the reads supporting the major allele; failure to meet this threshold resulted in assignment of an ambiguous base 'N' for both variant or invariant alleles with respect to the Nichols reference genome. Full genome alignment for these 29 genomes with respect to the Nichols reference and the SNP alignment from concatenated variant positions were generated. A phylogenetic tree was built in RAxML[21], using the maximum likelihood approach with a GTR+GAMMA substitution model and eight gamma categories of evolutionary rates for 1000 bootstrap replicates. ClonalFrameML[22] was used to detect possible sites of recombination from a full genome alignment. The maximum likelihood (ML) tree is provided as a clonal genealogy. We identified homoplastic SNPs between the yaws clade and the Bejel/SS14 clades by filtering for positions were the SS14 and/or the Bejel clade show the derived allele and at least one yaws strain shows the ancestral allele, while at least one other yaws strain shares the derived allele. We identified 13 positions that fulfill these criteria. These positions, in addition to those in recombinant positions identified by ClonalFrame, were excluded from further analysis (https://github.com/AdityaLankapalli/Recombination_tools). Positions with missing data ('N') were also removed. For these two datasets, ML trees were constructed as described above and trees were visualized (Figure 6).

SNP effects

The *Y. pestis* SNPs were called using UnifiedGenotyper in the Genome Analysis Toolkit (GATK) in EAGER, using the emit all sites option[23]. The resulting vcf file was processed with MultiVCFAnalyzer v0.85 (https://github.com/alexherbig/MultiVCFAnalyzer), to produce a SNP table[24]. Two hundred and seventy-five (275) genomes, consisting of both ancient and modern varieties, were used in the analysis, [25-36] (Table S15). MultiVCFAnalyzer parameters

included a minimum SNP coverage of 3-fold, genotyping quality of 30, and homozygosity calling at 90% support. An N was inserted in positions where no base call could be made based on the above parameters. Excluded regions are described elsewhere[37]. A total of 6,949 variant positions were identified in this set (Table S16). The unique variants associated with AGU025 were analyzed using SnpEff[38] (Tables S17 and S18). The 4 SNPs in AGU025 affected four different proteins. Two of these changes, at positions 4,047,235 and 4,171,875, affecting the *yjcD* and *aceB* genes respectively, were synonymous. The SNP at position 3,789,518, present in only AGU025, affected the *mrcB* gene, which is a penicillin binding protein. The SNP at position 4,232,523 affects the *fre* gene - NADPH (FMN reductase) and is present in both AGU025 and AGU020. One unique SNP in AGU025 at position 1577025 was disregarded following visual inspection: It was restricted to the terminal ends of reads at the edge of an uncovered region of the genome, and hence is more likely to result from mismapping than actual biological variation.

The above procedures were followed for the treponemal dataset with 31 genomes (Table S19), with exclusion of SNPs filtered for sites identified as recombinant (see above). The unique variants observed in AGU007 were analyzed using SnpEff version 4.3t. The variant type, its annotation, and effect were estimated using SnpSift and snpEff respectively. The two unique variants 39267 and 523975 cause non-synonymous changes in the amino acid residues of genes TPANIC_RS00150 (molecular chaperone GroEL), TPANIC_RS02370 (methyl-accepting chemotaxis protein) respectively.

Dating of the Yaws Clade

As input for TempEst, we used a maximum likelihood tree generated using RAxML[39] and an AGU007 tip date of 1464 CE (oldest sigma 2 values as determined from radiocarbon dating). The relationship between root-to-tip distance and date yielded an R^2 of approximately 0.053 (Figure S7). The MEGA maximum likelihood clock test was configured using MEGAproto with a general time reversible (GTR) substitution model with 4 gamma categories

The BEAST model used for molecular dating was configured with BEAUti[40]. Tip sampling for AGU007 and correction for static positions in the genome was enabled through manual editing of the configuration file. The best-fitting substitution model for the dataset as calculated by ModelGenerator[41] was the transversion model (TVM), which assumes variable base frequencies, variable transversion rates, and equal transition rates. This was implemented in BEAUti by selecting the GTR model and fixing the AG operator, with a relaxed clock and constant coalescent tree model.

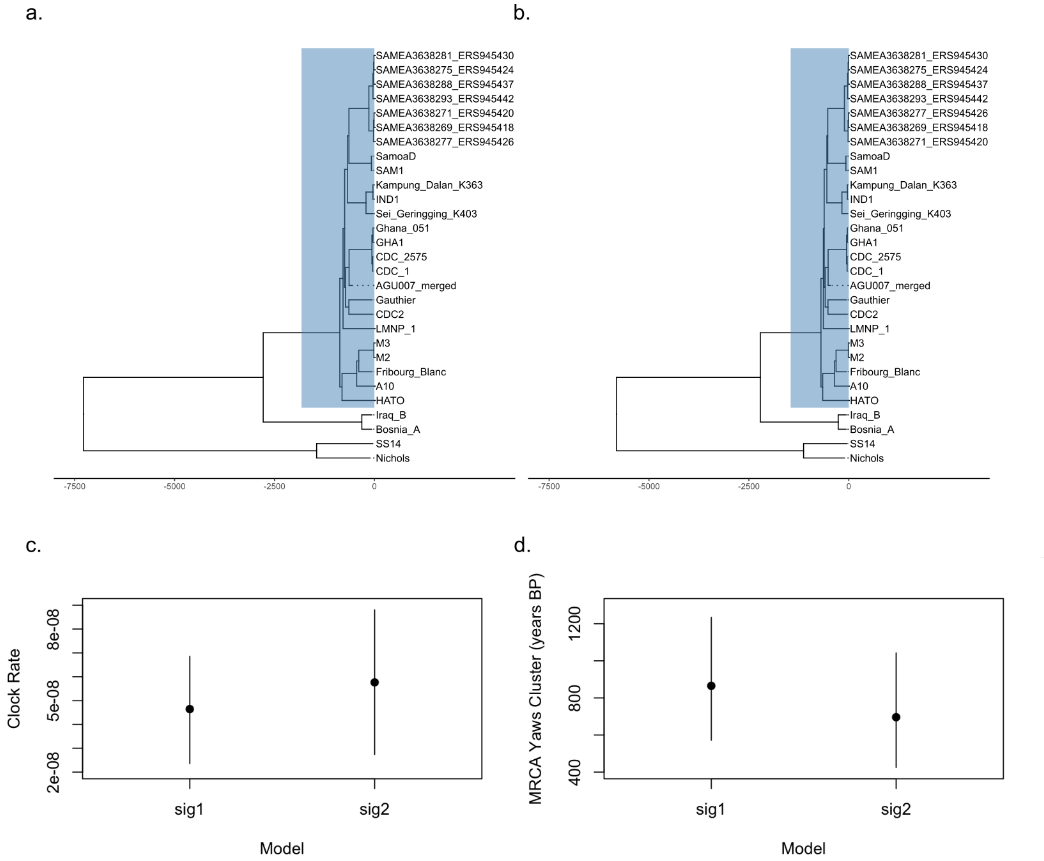

Figure S8: Molecular dating of yaws cluster with BEAST. a) MCC tree for sig1 model with yaws cluster. b) MCC tree for sig2 model. In both panel (a) and panel (b) the yaws cluster (*Treponema pallidum pertenue*) is highlighted in blue and the temporal scale at the bottom of each tree is in years before present. c) Comparison of clock rate estimates between models sig1 and sig2. The black dots indicate the estimated mean clock rate across the tree for each model, and the black lines represent the 95% HPD interval for each model. The y-axis is in substitutions per site per year. d) Comparison of the MRCA date for the yaws cluster according to each model. The black dots indicate the mean MRCA date, and the black lines represent the 95% HPD interval of the date for each model.

References cited

1 Dabney, J. *et al.* Complete mitochondrial genome sequence of a Middle Pleistocene cave bear reconstructed from ultrashort DNA fragments. *Proceedings of the National Academy of Sciences* **110**, 15758, doi:10.1073/pnas.1314445110 (2013).

2 King, C. E., Debruyne, R., Kuch, M., Schwarz, C. & Poinar, H. N. A quantitative approach to detect and overcome PCR inhibition in ancient DNA extracts. *BioTechniques* **47**, 941-949, doi:10.2144/000113244 (2009).

3 Schuenemann, V. J. *et al.* Targeted enrichment of ancient pathogens yielding the pPCP1 plasmid of *Yersinia pestis* from victims of the Black Death. *Proceedings of the National Academy of Sciences* **108**, E746, doi:10.1073/pnas.1105107108 (2011).

4 Meyer, M. & Kircher, M. Illumina sequencing library preparation for highly multiplexed target capture and sequencing. *Cold Spring Harb Protoc* **2010**, pdb.prot5448, doi:10.1101/pdb.prot5448 (2010).

5 Briggs, A. W. *et al.* Removal of deaminated cytosines and detection of in vivo methylation in ancient DNA. *Nucleic Acids Research* **38**, e87-e87, doi:10.1093/nar/gkp1163 (2009).

6 Rohland, N., Harney, E., Mallick, S., Nordenfelt, S. & Reich, D. Partial uracil–DNA–glycosylase treatment for screening of ancient DNA. *Philosophical Transactions of the Royal Society B: Biological Sciences* **370**, 20130624, doi:10.1098/rstb.2013.0624 (2015).

7 Andrades Valtueña, A. *et al.* The Stone Age Plague and Its Persistence in Eurasia. *Current Biology* **27**, 3683-3691.e3688, doi:https://doi.org/10.1016/j.cub.2017.10.025 (2017).

8 Peltzer, A. *et al.* EAGER: efficient ancient genome reconstruction. *Genome Biology* **17**, 60, doi:10.1186/s13059-016-0918-z (2016).

9 Lindgreen, S. AdapterRemoval: easy cleaning of next-generation sequencing reads. *BMC Research Notes* **5**, 337, doi:10.1186/1756-0500-5-337 (2012).

10 Jónsson, H., Ginolhac, A., Schubert, M., Johnson, P. L. F. & Orlando, L. mapDamage2.0: fast approximate Bayesian estimates of ancient DNA damage parameters. *Bioinformatics* **29**, 1682-1684, doi:10.1093/bioinformatics/btt193 (2013).

11 Parkhill, J. *et al.* Genome sequence of Yersinia pestis, the causative agent of plague. *Nature* **413**, 523-527, doi:10.1038/35097083 (2001).

12 Hübler, R. et al. HOPS: automated detection and authentication of pathogen DNA in archaeological remains. Genome Biology 20, 280, doi:10.1186/s13059-019-1903-0 (2019).

13 Herbig, A. *et al.* MALT: Fast alignment and analysis of metagenomic DNA sequence data applied to the Tyrolean Iceman. *bioRxiv*, 050559, doi:10.1101/050559 (2016).

14 Pětrošová, H. *et al.* Resequencing of Treponema pallidum ssp. pallidum Strains Nichols and SS14: Correction of Sequencing Errors Resulted in Increased Separation of Syphilis Treponeme Subclusters. *PLOS ONE* **8**, e74319, doi:10.1371/journal.pone.0074319 (2013).

15 Fu, Q. *et al.* DNA analysis of an early modern human from Tianyuan Cave, China. *Proceedings of the National Academy of Sciences* **110**, 2223, doi:10.1073/pnas.1221359110 (2013).

16 Arora, N. *et al.* Origin of modern syphilis and emergence of a pandemic Treponema pallidum cluster. *Nat Microbiol* **2**, 16245, doi:10.1038/nmicrobiol.2016.245 (2016).

17 Marks, M. *et al.* Diagnostics for Yaws Eradication: Insights From Direct Next-Generation Sequencing of Cutaneous Strains of Treponema pallidum. *Clin Infect Dis* **66**, 818-824, doi:10.1093/cid/cix892 (2018).

18 Knauf, S. *et al.* Nonhuman primates across sub-Saharan Africa are infected with the yaws bacterium Treponema pallidum subsp. pertenue. *Emerg Microbes Infect* **7**, 157, doi:10.1038/s41426-018-0156-4 (2018).

19 Peltzer, A. *et al.* EAGER: efficient ancient genome reconstruction. *Genome Biol* **17**, 60, doi:10.1186/s13059-016-0918-z (2016).

20 DePristo, M. A. *et al.* A framework for variation discovery and genotyping using next-generation DNA sequencing data. *Nat Genet* **43**, 491-498, doi:10.1038/ng.806 (2011).
21 Stamatakis, A. RAxML version 8: a tool for phylogenetic analysis and post-analysis of large phylogenies. *Bioinformatics* **30**, 1312-1313, doi:10.1093/bioinformatics/btu033 (2014).
22 Didelot, X. & Wilson, D. J. ClonalFrameML: efficient inference of recombination in whole bacterial genomes. *PLoS Comput Biol* **11**, e1004041, doi:10.1371/journal.pcbi.1004041 (2015).
23 DePristo, M. A. *et al.* A framework for variation discovery and genotyping using next-generation DNA sequencing data. *Nature Genetics* **43**, 491, doi:10.1038/ng.806 https://www.nature.com/articles/ng.806#supplementary-information (2011).
24 Bos, K. I. *et al.* Pre-Columbian mycobacterial genomes reveal seals as a source of New World human tuberculosis. *Nature* **514**, 494, doi:10.1038/nature13591 https://www.nature.com/articles/nature13591#supplementary-information (2014).
25 Bos, K. I. *et al.* A draft genome of *Yersinia pestis* from victims of the Black Death. *Nature* **478**, 506, doi:10.1038/nature10549 https://www.nature.com/articles/nature10549#supplementary-information (2011).
26 Feldman, M. *et al.* A High-Coverage *Yersinia pestis* Genome from a Sixth-Century Justinianic Plague Victim. *Molecular Biology and Evolution* **33**, 2911-2923, doi:10.1093/molbev/msw170 (2016).
27 Namouchi, A. *et al.* Integrative approach using *Yersinia pestis* genomes to revisit the historical landscape of plague during the Medieval Period. *Proceedings of the National Academy of Sciences* **115**, E11790, doi:10.1073/pnas.1812865115 (2018).
28 Eroshenko, G. A. *et al. Yersinia pestis* strains of ancient phylogenetic branch 0.ANT are widely spread in the high-mountain plague foci of Kyrgyzstan. *PLOS ONE* **12**, e0187230, doi:10.1371/journal.pone.0187230 (2017).
29 Chain, P. S. G. *et al.* Complete Genome Sequence of *Yersinia pestis*; Strains Antiqua and Nepal516: Evidence of Gene Reduction in an Emerging Pathogen. *Journal of Bacteriology* **188**, 4453, doi:10.1128/JB.00124-06 (2006).
30 Kislichkina, A. A. *et al.* Nineteen Whole-Genome Assemblies of *Yersinia pestis*, Including Representatives of Biovars caucasica, talassica, hissarica, altaica, xilingolensis, and ulegeica. *Genome Announcements* **3**, e01342-01315, doi:10.1128/genomeA.01342-15 (2015).
31 Rasmussen, S. *et al.* Early Divergent Strains of *Yersinia pestis* in Eurasia 5,000 Years Ago. *Cell* **163**, 571-582, doi:https://doi.org/10.1016/j.cell.2015.10.009 (2015).
32 Zhgenti, E. *et al.* Genome Assemblies for 11 *Yersinia pestis* Strains Isolated in the Caucasus Region. *Genome Announcements* **3**, e01030-01015, doi:10.1128/genomeA.01030-15 (2015).
33 Spyrou, M. A. *et al.* Phylogeography of the second plague pandemic revealed through analysis of historical *Yersinia pestis* genomes. *Nature Communications* **10**, 4470, doi:10.1038/s41467-019-12154-0 (2019).
34 Spyrou, Maria A. *et al.* Historical *Y. pestis* Genomes Reveal the European Black Death as the Source of Ancient and Modern Plague Pandemics. *Cell Host & Microbe* **19**, 874-881, doi:https://doi.org/10.1016/j.chom.2016.05.012 (2016).

35 Bos, K. I. *et al.* Eighteenth century *Yersinia pestis* genomes reveal the long-term persistence of an historical plague focus. *Elife* **5**, e12994-e12994, doi:10.7554/eLife.12994 (2016).

36 Spyrou, M. A. *et al.* Analysis of 3800-year-old *Yersinia pestis* genomes suggests Bronze Age origin for bubonic plague. *Nature Communications* **9**, 2234, doi:10.1038/s41467-018-04550-9 (2018).

37 Spyrou, M. A. *et al.* A phylogeography of the second plague pandemic revealed through the analysis of historical *Y. pestis* genomes. *bioRxiv*, 481242, doi:10.1101/481242 (2018).

38 Cingolani, P. *et al.* A program for annotating and predicting the effects of single nucleotide polymorphisms, SnpEff. *Fly* **6**, 80-92, doi:10.4161/fly.19695 (2012).

39 Stamatakis, A. RAxML version 8: a tool for phylogenetic analysis and post-analysis of large phylogenies. *Bioinformatics* **30**, 1312-1313, doi:10.1093/bioinformatics/btu033 (2014).

40 Bouckaert, R. *et al.* BEAST 2: A Software Platform for Bayesian Evolutionary Analysis. *PLOS Computational Biology* **10**, e1003537, doi:10.1371/journal.pcbi.1003537 (2014).

41 Keane, T. M., Creevey, C. J., Pentony, M. M., Naughton, T. J. & McLnerney, J. O. Assessment of methods for amino acid matrix selection and their use on empirical data shows that ad hoc assumptions for choice of matrix are not justified. *BMC Evolutionary Biology* **6**, 29, doi:10.1186/1471-2148-6-29 (2006).
